# Is pulmonary arterial hypertension associated with schistosomiasis distinct from pulmonary arterial hypertension associated with portal hypertension?

**DOI:** 10.1016/j.jhlto.2023.100007

**Published:** 2023-10-05

**Authors:** Brian B. Graham, Joan F. Hilton, Michael H. Lee, Rahul Kumar, Dara Fonseca Balladares, Farbod N. Rahaghi, Raúl San José Estépar, Claudia Mickael, Rodrigo Luís Barbosa Lima, Camila M.C. Loureiro, Juliana Lucena, Rudolf K.F. Oliveira, Ricardo de Amorim Corrêa

**Affiliations:** a Lung Biology Center, University of California San Francisco, San Francisco, California; b Pulmonary Division, San Francisco General Hospital, San Francisco, California; c Department of Epidemiology and Biostatistics, University of California San Francisco, San Francisco, California; d Pulmonary Medicine, Brigham and Women’s Hospital, Boston, Massachusetts; e Applied Chest Imaging Laboratory, Brigham and Women’s Hospital, Boston, Massachusetts; f Pulmonary and Critical Care Medicine, University of Colorado Anschutz Medical Campus, Aurora, Colorado; g Pulmonary Medicine, Hospital Júlia Kubitscheck, Belo Horizonte, Minas Gerias, Brazil; h Pulmonary Medicine, Santa Casa da Bahia, Salvador, Bahia, Brazil; i Division of Respiratory Diseases, Department of Medicine, Federal University of São Paulo, São Paulo, Brazil; j Internal Medicine/Pulmonary Division, Medical School, Federal University of Minas Gerais, Belo Horizonte, Minas Gerais, Brazil

**Keywords:** pulmonary arterial hypertension, schistosomiasis, portal hypertension, TGF-beta signaling, thromboembolism

## Abstract

Pulmonary arterial hypertension associated with schistosomiasis (SchPAH) and pulmonary arterial hypertension associated with portal hypertension (PoPAH) are lung diseases that develop in the presence of liver diseases. However, mechanistic pathways by which the underlying liver conditions and other drivers contribute to the development and progression of pulmonary arterial hypertension (PAH) are unclear for both etiologies. In turn, these unknowns limit certainty of strategies to prevent, diagnose, and reverse the resultant PAH. Here we consider specific mechanisms that contribute to SchPAH and PoPAH, identifying those that may be shared and those that appear to be unique to each etiology, in the hope that this exploration will both highlight known causal drivers and identify knowledge gaps appropriate for future research. Overall, the key pathophysiologic differences that we identify between SchPAH and PoPAH suggest that they are not variants of a single condition.

## Background—Why is this important?

Pulmonary arterial hypertension associated with schistosomiasis (SchPAH) and pulmonary arterial hypertension associated with portal hypertension (PoPAH) are both forms of pulmonary arterial hypertension (PAH), classified as group 1 by the 2022 European Society of Cardiology and European Respiratory Society Guidelines.^1^ In Western countries, PAH is rare, found in 15 to 50 persons per million within the United States (US) and Europe.^1^ In the US-based PVDomics cross-sectional survey,^2^ PoPAH and SchPAH accounted for 5% and < 3% of 353 PAH cases, whereas idiopathic PAH accounted for 45%. However, in tropical countries where schistosomiasis is endemic, SchPAH is likely to be more prevalent than in the US and Europe.

**Figure 1:**
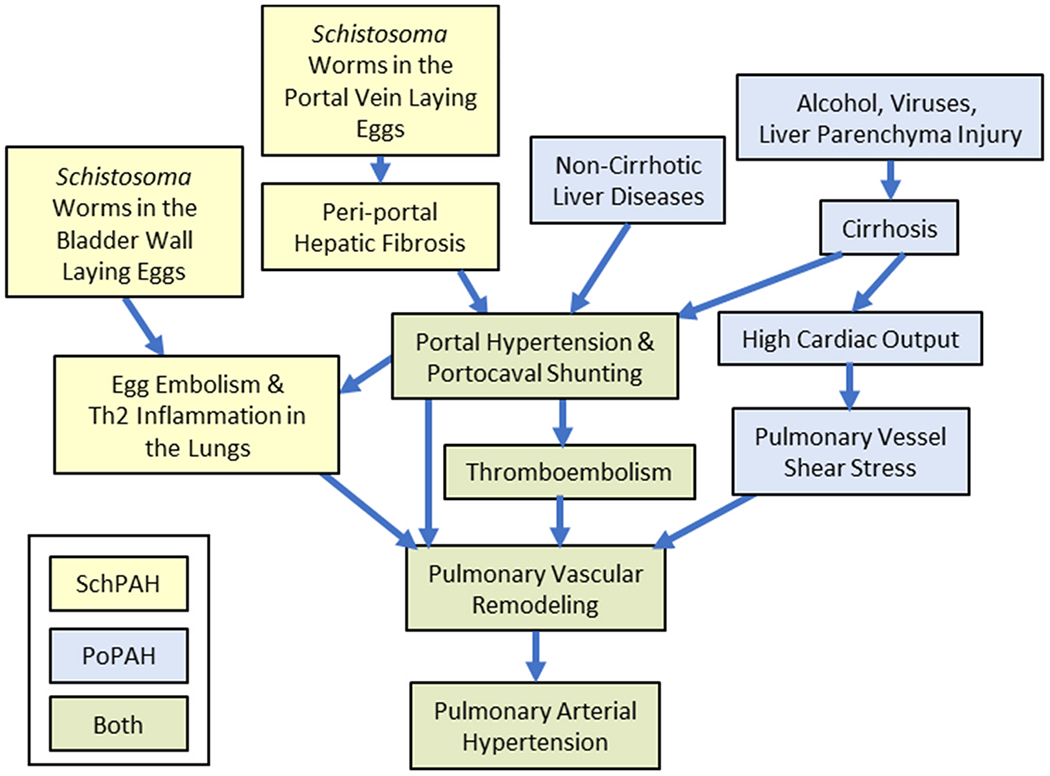
Proposed pathways of mechanisms underlying SchPAH (yellow), PoPAH (blue), or shared by both (green). PoPAH, pulmonary arterial hypertension associated with portal hypertension; SchPAH, pulmonary arterial hypertension associated with schistosomiasis.

Schistosomiasis results from infection by the trematode flatworm *Schistosoma*, a snail-borne parasite endemic in tropical regions of Africa, Brazil, the Middle East, and Southeast Asia. Over 250 million people worldwide have schistosomiasis.^3^ It is estimated that 0.25% to 1% of individuals with chronic schistosomiasis develop SchPAH.^4^ The regions where the parasitic infection is prevalent are often resource challenged, severely limiting effective diagnosis and treatment of PAH. After entry into the body, the worms migrate to the target organ (generally the portal vein) where they persist for years. A majority of the eggs erode through the intestinal wall to return to the environment and complete the lifecycle, but a minority of the ~100 μm diameter eggs are retained in the host, floating downstream to the liver, and causing granulomatous type 2 inflammation. About 10% of chronically infected individuals develop dense periportal fibrosis, termed schistosomiasis-associated hepatosplenic disease (SchHSD).^5^ SchHSD causes portal hypertension, portocaval shunting, and the transit of *Schistosoma* eggs to systemic veins, and then the lungs (Figure 1). The pulmonary pathology of SchPAH includes type 2 inflammation around the eggs,^6^ but otherwise, the pathology is similar to other PAH etiologies, including plexiform lesions.

PoPAH most commonly occurs in the setting of liver cirrhosis caused by alcohol, viral hepatitis, autoimmune hepatitis, and primary biliary cirrhosis. PoPAH can also occur in noncirrhotic portal hypertensive liver diseases, including nonalcoholic steatohepatitis,^7^ and primary sclerosing cholangitis^7,8^ which has more limited hepatocellular injury. In cirrhosis, decreased liver function reduces clearance of vasoactive substances including atrial natriuretic peptide, renin, angiotensin II, and aldosterone.^9^ This results in systemic vasodilation and increased cardiac output, which has been hypothesized to increase pulmonary vascular shear stress, resulting in endothelial cell injury and vascular remodeling.^10,11^ It is estimated 2% to 10% of those with portal hypertension develop PoPAH.^12^ Polymorphisms in estrogen metabolism genes and greater plasma levels of estrogen pathway metabolites correlate with the development of PoPAH in those at risk.^8,13^

A major knowledge gap in the PAH field is whether the liver disease present in both SchPAH and PoPAH, hepatosplenic schistosomiasis and most commonly cirrhosis, respectively, contributes to these pulmonary vascular diseases in the same manner. Patients with both SchPAH and PoPAH benefit from treatment with PAH-targeted therapies,^14,15^ but while these medications reduce symptoms and likely improve survival,^16–18^ they likely do not directly reverse the mechanisms that underlie the diseases, which are not well understood. Therefore, clarifying shared mechanisms will enhance understanding of the pathogenesis and effective clinical management of both etiologies.

## SchPAH and PoPAH commonalities

### Hepatocellular injury

SchHSD is generally not thought to have the same severity of liver dysfunction as cirrhosis, as less of the total parenchyma is fibrotic. Biochemical studies indicate the synthetic dysfunction is more severe among those with PoPAH than those with SchHSD.^7,19,20^ However, hepatocellular injury severity is not associated with the development of PoPAH (Table 1) and, more specifically, the Model for End-Stage Liver Disease score does not correlate with the incidence of PoPAH.^7,21^ Furthermore, PoPAH can complicate liver diseases that have relatively little hepatocellular dysfunction. In SchPAH, there are case reports of *S. haematobium* infection causing PAH^22^: *S. haematobium* worms home to the bladder wall, causing urinary disease without liver disease. Lastly, mice experimentally challenged with intraperitoneal *Schistosoma* egg sensitization followed by intravenous egg administration and embolism of the pulmonary arteries (PAs) develop pulmonary hypertension (PH), a model which has no liver disease at all.^23,24^ Overall, it is apparent that hepatocellular injury is shared by both SchPAH and PoPAH, but is actually unlikely to directly contribute to the development of either form of PAH.

### Portal hypertension and portosystemic shunts

The severity of portal hypertension is likely comparable between SchPAH and PoPAH, as the incidence of variceal bleeding is similar,^25–27^ despite the fibrosis distribution being periportal in SchHSD vs bridging in liver cirrhosis. Entirely aside from parenchymal forms of liver disease, portosystemic shunts resulting from congenital malformations are associated with PAH. Congenital extrahepatic portocaval shunt (CEPS) is caused by abnormal embryonic development of the portal vein, and many case series and case reports have described PAH occurring in the setting of CEPS.^28–30^ In summary, both portal hypertension and portosystemic shunts are shared between SchPAH and PoPAH, and are likely to contribute to the pathogenesis of both etiologies.

### Dysregulated transforming growth factor beta (TGF-β) signaling

Alterations in transforming growth factor beta (TGF-β) superfamily signaling underlies many PAH etiologies. Familial PAH most commonly results from bone morphogenetic protein (BMP) receptor type II loss of function, causing decreased BMP pathway signaling and a reciprocal increase in TGF-β pathway signaling. Patients with PoPAH have evidence of reduced BMP signaling, including less serum BMP9^31^ and increased endoglin^32^ (which functions as a BMP ligand trap,^33^ thereby decreasing BMP signaling). In comparison, SchPAH has increased TGF-β pathway signaling (which would reciprocally decrease BMP signaling), with higher serum TGF-β ^34^ and increased tissue phospho-Smad2/3 expression.^24^ In *Schistosoma*-exposed mice, blocking TGF-β signaling prevents PH.^23,24^ Overall, it is likely that a shift in TGF-β family signaling, with increased TGF-β pathway and less BMP pathway signaling, is a commonality in both SchPAH and PoPAH, and is likely to contribute to the pathogenesis of both.

### Thromboembolism

Thromboembolism likely contributes to all forms of PAH in varying degrees, related to a hypercoagulable state and slower blood flow through lung regions.^35^ In PoPAH, thrombi formed in the portal veins may travel through portocaval shunts to become pulmonary emboli, with relatively frequent pulmonary emboli described in this condition.^36,37^ Thromboembolic disease has also been reported in SchPAH.^38^ Patients with SchPAH can have aneurysmal proximate PAs, with mural thrombus, but it is not clear if there is also distal embolization in these cases. In summary, thromboembolism may be relatively common in and contribute to the development of both SchPAH and PoPAH, but here the evidence is not very clear, and there may be cases that develop in the absence of thromboemboli.

## SchPAH and PoPAH distinctions

### PoPAH: Higher cardiac output and endothelial shear stress

Increased cardiac output is observed in PoPAH,^7^ leading to shear stress on the pulmonary endothelium which may contribute to PoPAH pathogenesis.^10,11^ In contrast, patients with SchPAH have normal or low cardiac output,^39,40^ as seen in other (non-PoPAH) forms of PAH.

### SchPAH: Greater pulmonary inflammation

Perivascular inflammation is uncommon in PoPAH, although histologic series are limited.^36^ In contrast, perivascular inflammation is prominent, robust, and widely described in SchPAH, including T cells, mast cells, and dendritic cells,^41^ and markers of type 2 inflammation.^6^ Blocking type 2 inflammation and depleting T cells are protective in *Schistosoma*-PH mice.^6,42^ Inflammation of the pulmonary vasculature in SchPAH may contribute to proximal PA aneurysms,^43^ as is seen in other inflammatory conditions including Behçet disease and Hughes-Stovin syndrome.^44,45^

### Disease trajectories

Patients with PoPAH who have not undergone transplantation have a prognosis worse than prototypical idiopathic PAH,^46^ whereas patients with SchPAH are thought to have a prognosis better than idiopathic PAH.^15^

Remarkably, the relative trajectories reverse when the underlying liver condition is treated. Patients with PoPAH who undergo liver transplantation almost universally have some degree of improvement.^47,48^ These data suggest that in PoPAH, the driving agent is fundamentally the liver, as replacing the liver results in pulmonary vascular recovery. A potential mechanistic explanation is when the cardiac output normalizes, the endothelial shear stress also normalizes, and the pulmonary vasculature can heal. Reversing pulmonary vascular pathology by unloading is also described in humans with PAH associated with congenital heart disease,^50^ and in animal models.^49^

In patients with SchHSD, antihelminthic treatment with praziquantel results in less liver fibrosis^51^ and reduced portal hypertension.^52^ However, patients with SchPH treated with praziquantel do not appear to have improvement in their lung disease.^53,54^ Parasite eradication may slow the rate of progression by stopping further egg deposition in the lungs, but patients still die of SchPAH despite receiving praziquantel, avoiding reinfection by moving away from endemic settings, and not having any eggs in their lungs at the time of death.^38^ These data suggest that in SchPAH, after a certain point, the liver pathology and the lung pathology become disconnected: the liver improves but the lung does not. Potentially the lung inflammation initially triggered by *Schistosoma* eggs results in persistent pulmonary vascular pathology, now independent of either the parasite or the liver disease. However, these observations may be confounded by less access to care including liver transplantation, more advanced disease, and the potential for chronic reinfection in endemic settings.

## Summary and future directions

Overall, we suggest that PoPAH and SchPAH are unlikely to arise from the same pathologic mechanisms, and SchPAH should not be considered a variant of PoPAH. It is likely that both portosystemic shunts and abnormal TGF-β signaling contribute to the pathology of both PoPAH and SchPAH, whereas the hepatocellular injury present in both is unlikely to contribute to either. Increased vascular shear stress from increased cardiac output probably uniquely contributes to PoPAH and reverses at least in part by liver transplantation. In contast, the localized type 2 inflammation arising from egg embolization is a unique contributor to SchPAH.

There remain open questions about the overlap between the 2 conditions (Table 2). We suggest that identifying molecular pathways that are shared by the 2 etiologies increases the probability that these are underlying disease drivers, and if targeted would be more likely to have clinical benefit.

It would be useful to assess the same plasma and lung tissue biomarkers in both conditions to identify potentially shared cellular pathways. It would also be helpful to have better tools to quantify the degree of exposure over time, such as the burdens of parasitic and/or viral infection in SchPAH and cirrhosis in PoPAH, respectively. Genetic variants and environmental exposure factors likely contribute to the development of both SchPAH and PoPAH in at-risk individuals. An important environmental exposure is the potential for coinfection, such as with HIV which can independently cause PAH, as the combination is likely to result in synergistic PAH pathobiology.^55^

Imaging studies would help characterize SchPAH and PoPAH pathology noninvasively, including computed tomography imaging to assess the pulmonary vasculature, and ventilation perfusion study (V/Q) imaging to identify thromboembolic disease. In experimental animal models of each condition, interrogating whether targeting the same pathways can suppress PH will identify candidate shared pathophysiologic mechanisms. Ultimately, clinical trials targeting pathogenic mechanisms need to be performed. As TGF-β family signaling likely underlies both conditions, we hypothesize the activin/growth differentiation factor ligand trap sotatercept will work in both PoPAH and SchPAH, as it has shown great promise in clinical trials,^56,57^ but has not yet been studied in either etiology.

## Disclosure statement

The authors declare the following financial interests/personal relationships which may be considered as potential competing interests: Brian Graham reports financial support was provided by National Institutes of Health. Claudia Mickael reports financial support was provided by National Institutes of Health. Camila Melo Coelho Loureiro reports financial support was provided by Janssen-Cilag Pharmaceutical. Camila Melo Coelho Loureiro reports financial support was provided by Bayer SA. Dara Fonseca Balladares reports financial support was provided by National Institutes of Health. Farbod N Rahaghi reports financial support was provided by National Institutes of Health. Joan F. Hilton reports financial support was provided by National Institutes of Health. Michael Lee reports financial support was provided by National Institutes of Health. Michael Lee reports financial support was provided by Fulbright US Scholar Program. Michael Lee reports financial support was provided by US Department of Defense. Rahul Kumar reports financial support was provided by American Heart Association Inc. Rahul Kumar reports financial support was provided by American Thoracic Society. Rahul Kumar reports financial support was provided by The Cardiovascular Medical Research and Education Fund. Rahul Kumar reports financial support was provided by United Therapeutics Corporation. Ricardo Correa reports financial support was provided by Janssen-Cilag Farmacêutica. Ricardo Correa reports financial support was provided by Bayer SA. Raul San Jose Estepar reports financial support was provided by National Institutes of Health. Raul San Jose Estepar reports financial support was provided by Lung Biotechnology PBC. Raul San Jose Estepar reports financial support was provided by Insmed Inc. Raul San Jose Estepar reports financial support was provided by Boehringer Ingelheim GmbH. Raul San Jose Estepar reports financial support was provided by IMBiotechnologies Ltd. Raul San Jose Estepar reports financial support was provided by Leuko Labs. Raul San Jose Estepar reports financial support was provided by Chiesi Pharmaceuticals GMBH. Rudolf Oliveira reports financial support was provided by National Council for Scientific and Technological Development (CNPq, Brazil). Rodrigo Lima reports financial support was provided by GSK Brazil.

## Figures and Tables

**Table 1:** Summary of Pathophysiologic Mechanisms That are Likely to be Shared by SchPAH and PoPAH, or Unique to SchPAH or to PoPAH

Potential mechanisms		Relevant in SchPAH?	Relevant in PoPAH?
Liver-specific	Hepatocellular injury	Unlikely	Unlikely
	Portal hypertension	Likely	Likely
	Portocaval shunting	Likely	Likely
Lung-specific	↓ BMP / ↑ TGF-β signaling	Likely	Likely
	Egg embolization	Likely	No
	Type 2 inflammation	Likely	Unlikely
Circulating factors	Altered vasoactive metabolite concentrations	Unknown	Likely
	↑ Estrogen	Unknown	Likely
	↓ BMP9	Unknown	Likely
	↑ TGF-β	Likely	Unknown
Thromboembolism	Thromboemboli	Possibly	Possibly
Cardiac and shear stress	↑ Cardiac output	Unlikely	Likely
	Pulmonary vessel shear stress	Unlikely	Likely

BMP, bone morphogenetic protein; TGF-β, transforming growth factor beta.

**Table 2:** Proposed Questions for Future Research Comparing SchPAH and PoPAH, and Potential Approaches That May Be Considered

Questions for future research	Potential approaches
Are circulating biomarkers shared or different? - Estrogen metabolism - TGF-β family signaling - Type 2 inflammation - Markers of liver injury	Assess candidates in plasma samples from patients with SchPAH and PoPAH - Estrogen - BMP9, soluble endoglin, TGF-β - IL-4, IL-13 - Vasoactive substances - Higher throughput ‘omics approaches
Is there evidence of similar inflammation in PoPAH and SchPAH lung tissue?	Analyze autopsy or explanted lung tissue - Cytokines: IL-4, IL-13, TGF-β family - Cells: eosinophils, macrophages, and cell phenotypes
Does blocking the same pathways inhibit animals models of the diseases? - Does increasing BMP signaling protect in SchPAH? - Does blocking TGF-β or type 2 inflammation protect in PoPAH?	Interrogate animal models - Test if increasing BMP9 inhibits experimental SchPAH - Test if inhibiting TGF-β or IL-4/IL-13 prevents experimental PoPAH
Do patients with SchPAH and PoPAH have similar vascular morphology?	Compare pulmonary vasculature by etiology - Morphology based on CT imaging - Morphometric and stereologic assessments of lung tissue
Do patients with SchPAH and PoPAH have comparable portal hypertension severity? Does portal hypertension severity correlate with PAH development?	Assessment of portal pressure - Directly by hemodynamics - Indirectly such as by assessing portal vein diameter or spleen volume on CT imaging - Assessing patients with SchPAH and PoPAH, as compared to at-risk but PAH negative patients (SchHSD and cirrhosis, respectively)
Is thromboembolic disease a contributor to both SchPAH and PoPAH?	Systematic comparisons using CT and V/Q imaging
What are environmental risk factors for the development of SchPAH and PoPAH? - Coinfections, such as HIV - Pollution or toxins including alcohol	- Human epidemiologic studies to detect risk factors - Animal models with coexposure approaches
Are there shared genetic risk factors for the development of SchPAH and PoPAH?	Targeted genetic studies in both cohorts (particularly in SchPAH, where this has not been done yet)

BMP, bone morphogenetic protein; CT, computed tomography; PAH, pulmonary arterial hypertension; PoPAH, pulmonary arterial hypertension associated with portal hypertension; SchHSD, schistosomiasis-associated hepatosplenic disease; SchPAH, pulmonary arterial hypertension associated with schistosomiasis; TGF, transforming growth factor; V/Q, ventilation perfusion study.
